# Thyroid Endocrine Disruption in Zebrafish Larvae after Exposure to Mono-(2-Ethylhexyl) Phthalate (MEHP)

**DOI:** 10.1371/journal.pone.0092465

**Published:** 2014-03-21

**Authors:** Wenhui Zhai, Zhigang Huang, Li Chen, Cong Feng, Bei Li, Tanshi Li

**Affiliations:** 1 Department of Emergency, Chinese PLA General Hospital, Beijing, P.R. China; 2 Department of Emergency, the 305 Hospital of PLA, Beijing, P.R. China; Institut de Génomique Fonctionnelle de Lyon, France

## Abstract

Phthalates are extensively used as plasticizers in a variety of daily-life products, resulting in widespread distribution in aquatic environments. However, limited information is available on the endocrine disrupting effects of phthalates in aquatic organisms. The aim of the present study was to examine whether exposure to mono-(2-ethylhexyl) phthalate (MEHP), the hydrolytic metabolite of di-(2-ethylhexyl) phthalate (DEHP) disrupts thyroid endocrine system in fish. In this study, zebrafish (*Danio rerio*) embryos were exposed to different concentrations of MEHP (1.6, 8, 40, and 200 μg/L) from 2 h post-fertilization (hpf) to 168 hpf. The whole-body content of thyroid hormone and transcription of genes involved in the hypothalamic-pituitary-thyroid (HPT) axis were examined. Treatment with MEHP significantly decreased whole-body T4 contents and increased whole-body T3 contents, indicating thyroid endocrine disruption. The upregulation of genes related to thyroid hormone metabolism (*Dio2* and *UGT1ab*) might be responsible for decreased T4 contents. Elevated gene transcription of *Dio1* was also observed in this study, which might assist to degrade increased T3 contents. Exposure to MEHP also significantly induced transcription of genes involved in thyroid development (*Nkx2.1* and *Pax8*) and thyroid hormone synthesis (*TSHβ*, *NIS* and *TG*). However, the genes encoding proteins involved in TH transport (transthyretin, *TTR*) was transcriptionally significantly down-regulated after exposure to MEHP. Overall, these results demonstrate that acute exposure to MEHP alters whole-body contents of thyroid hormones in zebrafish embryos/larvae and changes the transcription of genes involved in the HPT axis, thus exerting thyroid endocrine toxicity.

## Introduction

Phthalate esters are a group of industrial chemicals extensively used as plasticizers in a variety of commercial products, such as polyvinyl chloride (PVC) floors, food packaging, clothing, toys, films, paints, adhesives, lubricants, cosmetics, electronics, ink printers and biomedical devices (e.g., blood transfusion bags) [1], [2]. A recent study estimated that the worldwide production of phthalates reached 11 billion pounds every year [3]. Because they are not chemically bonded to the polymer, phthalates tend to release from the matrix with time and use [4]. Various phthalate esters have been detected in all environmental compartments, including indoor and ambient air, indoor dust, water sources, and sediments [5]. Moreover, phthalates have tendency to bioaccumulate in fish and, eventually entering the food chain [6].

Of the various phthalates, di-(2-ethylhexyl) phthalate (DEHP) is one of the most frequently used phthalates, and it accounts for approximately 50% of total plasticizer production [7]. Due to its large-scale production and widespread use, the general population is continuously exposed to DEHP, mainly via inhalation, ingestion, dermal contact and intravenous routine throughout their whole lifetime, including in the intrauterine environment during pregnancy [8], [9]. Once enters into human body, DEHP is readily metabolized by esterases in the gut to mono-(2-ethylhexyl) phthalate (MEHP), which is more toxic than its parent compound [2]. In addition, MEHP might also be formed out of DEHP by abiotic and biotic processes in natural environment [10]–[13]. For example, MEHP has been detected in intravenous solutions stored in medical grade PVC bags [11], serum and plasma products packed into plastic containers and water from medical grade PVC tubing [12]. Therefore, MEHP is an environmental contaminant of its own, and have been detected in many environmental media. For instance, Suzuki et al., observed MEHP in the Tama River in Tokyo at concentrations of 0.01–1.3 μg/L (2001). A recent study demonstrated that MEHP was detected in all sediment and soil samples ranged from 13.0 to 166.7 ng/g [14]. Moreover, MEHP has been widely detected in human biological samples such as milk, urine, saliva, and serum [15]–[19]. Therefore, the potential health risks caused by DEHP/MEHP are receiving growing public concern.

Since thyroid hormones (THs) are essential for fetal development of the brain and involved in numerous physiological processes, the impact of environmental chemicals on the thyroid endocrine system has received increasing attention in recent years [20]. Chemicals may exert thyroid disrupting effects through disturbing the THs synthesis, secretion, transport, binding, action, or elimination [21]. Studies investigating the association between exposure to DEHP/MEHP and thyroid function are limited. An *in vitro* study demonstrated that DEHP enhances iodine uptake by modulating the sodium/iodide symporter in rat thyroid cell line [22]. In animal studies, rats administered diets containing DEHP showed lower plasma thyroxine (T4) and histological changes in the thyroid such as reduced follicle size and colloid density, [23]–[26], while rats intravenously receiving DEHP displayed increased concentrations of serum triiodothyronine (T3) and T4 [27]. In humans, increased urinary concentrations of MEHP were associated with decreased concentrations of total T3 and free T4 in the serum of adult men [28], [29]. Similar results were also found in a cross-sectional study conducted in Danish children, which demonstrated that urinary DEHP metabolites were inversely related to total and free T3 levels [30].

Thyroid endocrine disruption are of particular concern to amphibians and teleosts as these animals may be exposed to waterborne contaminants during a portion or the entirety of their life span. In teleosts and amphibians, the homeostasis of circulating THs is regulated through a finely tuned negative feedback mechanism by the hypothalamic-pituitary-thyroid (HPT) axis [31]. In many aspects, the thyroid system of zebrafish is similar to the mammalian or the amphibian thyroid system, and zebrafish are widely used as a vertebrate model in several areas of research with the prospect of extrapolating findings to other vertebrates and humans [32]–[35]. It has been shown that developing zebrafish embryos/larvae were a reliable model to assess chemical disruption of the thyroid endocrine system [36]–[38]. However, the potential thyroid endocrine disrupting effects of MEHP in fish, especially during the early developmental stages has not been fully elaborated up to now. In the present study, we investigated the effects of MEHP on mRNA expression involved in the HPT axis using zebrafish embryos/larvae. Moreover, an enzyme-linked immunosorbent assay (ELISA) was employed to measure whole-body thyroid hormone contents after MEHP exposure.

## Materials and Methods

### Chemicals

MEHP (CAS No: 4670-20-9, 99%) was purchased from AccuStandard (New Haven, CT, USA). Stock solution of MEHP was prepared by dilution in dimethyl sulfoxide (DMSO) (purity>99%), which was obtained from Amresco (Solon, OH, USA), and was stored at 4°C. 3-Aminobenzoic acid ethyl ester or methane-sulfonate salt (MS-222) was obtained from Sigma (St. Louis, MO, USA). All other chemicals used in this study were of analytical grade.

### Fish maintenance and experimental design

Adult zebrafish (*Danio rerio*) of the wild-type (AB strain) were maintained in a flow-through system in charcoal-filtered tap water at a constant temperature (28±1°C), with a photoperiod 14∶10 (light:dark). Fish were fed a commercial food pellet (Trea, Germany) and newly hatched brine shrimp (*Artemia nauplii*) two times daily in a quantity that was consumed within 5 minutes. Zebrafish embryos were obtained from spawning adults in a ratio of 2∶1 (male: female) in tanks overnight. Spawning was triggered in the morning when the light was turned on and was usually completed within 30 min. At 2 h post-fertilization (hpf), embryos were examined under a dissecting microscope, and those embryos that had developed normally and reached the blastula stage were selected for subsequent experiments. Fertilization rate of the batch of eggs used was at least 90%. Normal embryos (approximately 400) were randomly distributed into separate glass beakers containing 500 mL of exposure solution (0, 1.6, 8, 40, and 200 μg/L MEHP) containing 0.2 mM Ca (NO_3_)_2_, 0.13 mM MgSO_4_, 19.3 mM NaCl, 0.23 mM KCl and 1.67 mM HEPES [39] until 168 hpf. The selected exposure concentrations were previously ascertained by performing a range-finding study, and the lowest concentration employed in this study was based on an environmental investigation [10]. During the experimental period, the exposure solutions were renewed daily, and both the control and exposure groups with 6 replicates in each exposure concentration were treated with 0.01% (v/v) DMSO. After exposure, zebrafish larvae were anesthetized in 0.03% MS-222 and randomly sampled for body length and weight determination, mRNA expression analysis and thyroid hormones measurement. The hatching, malformation, growth and survival were also recorded.

### Ethics Statement

This study was approved by the Institutional Animal Care and Use Committee (IACUC) at the 305 Hospital of PLA of China.

### Thyroid hormone extraction and measurement

Procedures for thyroid hormone extraction and measurement were performed as described by Yu et al. [38] and Wang et al. [40]. Briefly, approximately 200 larvae for each treatment were homogenized in 0.2 mL ELISA buffer provided in the kits. The samples were then disrupted for 10 min on ice by spasmodic sonication. After centrifugation at 5000 × g for 10 min at 4°C, the supernatants were collected for T3 and T4 measurement using commercial ELISA kits (Uscnlife, Wuhan, China) following the manufacture's instructions. The detection limits for T3 and T4 were 0.1 ng/mL and 1.2 ng/mL, respectively. Intra-assay and inter-assay variations were below 15% in this study. No significant cross-reactivity or interference was observed for each kit.

### RNA isolation and quantitative real-time polymerase chain reaction (qRT-PCR)

Total RNA was isolated from 30 homogenized larvae using RNAiso Plus reagent (Takara Biochemicals, Dalian, China), all procedures followed the manufacturer's instruction and the published protocols. The concentration of total RNA was measured at 260 nm using a NanoDrop ND-2000 spectrophotometer (Thermo Scientific, Wilmington, DE). The RNA quality was examined by measuring the 260/280 nm ratios and 1% agarose-formaldehyde gel electrophoresis with ethidium bromide staining. Approximately 1 μg total RNA was retrotranscribed by PrimeScript RT reagent Kit with gDNA Eraser (Perfect Real Time) (Takara Biochemicals, Dalian, China) according to the manufacturer's instructions. Quantitative real-time PCR was analyzed on an ABI 7300 System (PerkinElmer Applied Biosystems, Foster City, CA, USA) using SYBR Green PCR kit (Takara Biochemicals, Dalian, China). The primer sequences of the selected genes were obtained by using the online Primer 3 program (http://frodo.wo.mit.edu/) and are listed in **Table 1**. The PCR reaction comprised an initial denaturation step at 95 °C for 30 s, followed by 40 cycles at 95 °C for 5 s, 60 °C for 15 s, and 72 °C for 45 s. A melting temperature-determining dissociation step was performed at 95 °C for 30 s, 57 °C for 30 s, and 72 °C for 60 s at the end of the amplification phase. The dissociation curve was used to check the specificity of PCR products. All the samples were analyzed in triplicate and the mean value of these triplicate measurements were used for the calculations of the mRNA transcriptions. The housekeeping gene ribosomal protein L8 (*rpl8*) did not vary upon chemical exposure (data not shown) and was used as internal control. The expression levels of genes were normalized to *rpl8* mRNA contents using the 2^−ΔΔCt^ method.

**Table 1:** Primer sequences for the genes tested in the present study.

Gene name	Primer Sequence (5′-3′)		Accession Number
	Forward	Reverse	
*Rpl8*	ttgttggtgttgttgctggt	ggatgctcaacagggttcat	NM_200713
*TSHβ*	gcagatcctcacttcacctacc	gcacaggtttggagcatctca	AY135147
*Nkx2.1a*	aggacggtaaaccgtgtcag	caccatgctgctcgtgtact	NM_131589
*Pax8*	gaagatcgcggagtacaagc	ctgcactttagtgcggatga	AF072549
*NIS*	ggtggcatgaaggctgtaat	gatacggcatccattgttgg	NM_001089391
*TG*	ccagccgaaaggatagagttg	atgctgccgtggaatagga	XM_001335283
*TTR*	cgggtggagtttgacacttt	gctcagaaggagagccagta	BC081488
*Dio1*	gttcaaacagcttgtcaaggact	agcaagcctctcctccaagtt	BC076008
*Dio2*	gcataggcagtcgctcattt	tgtggtctctcatccaacca	NM_212789
*UGT1ab*	ccaccaagtctttccgtgtt	gcagtccttcacaggctttc	NM_213422

### Statistical analysis

Normality and homogeneity of data were analyzed using the Kolmogorov-Smirnov test, and Levene's test, respectively. All data were shown as means ± standard error (SEM). One-way analysis of variance (ANOVA) was applied to calculate statistical significance followed by Dunnett's test as a post hoc test to independently compared each exposure group to the control group. The LSD test was used as a post hoc test for multiple comparisons between groups. For hatching, survival and malformation rates, as these data were presented as proportions, they were square root arcsine-transformed before analysis of variance. All the analyses were conducted with SPSS statistical software version 13.0 (SPSS, Inc., Chicago, IL, USA). A *p* < 0.05 was considered statistically significant.

## Results

### Developmental toxicity

There were no significant effects on hatching, malformation, survival, body length and weight after exposure to MEHP (1.6, 8, 40 and 200 μg/L) relative to the control until 168 hpf **(** **Table 2** **)**.

**Table 2:** Development index of zebrafish larvae after exposure to MEHP (0, 1.6, 8, 40 and 200 μg/L) until 168 hpf.^a^

MEHP (μg/L)	0	1.6	8	40	200
Hatching^a^ (%)	93.5±1.2	91.9±2.6	94.4±0.7	90.8±0.7	93.2±0.4
Malformation^a^ (%)	2.08±0.42	3.02±0.87	2.04±0.43	2.57±0.25	3.01±0.38
Survival^a^ (%)	75.2±1.3	73.2±3.4	76.5±1.0	72.6±0.1	74.9±0.2
Length^a^ (mm)	3.41±0.13	3.41±0.05	3.52±0.11	3.32±0.06	3.11±0.13
Weight^a^ (mg)	0.36±0.03	0.37±0.01	0.36±0.03	0.35±0.04	0.34±0.01

a The values represent mean ± standard error (SEM) of six replicate groups.

### Whole-body TH content

**Figure 1:**
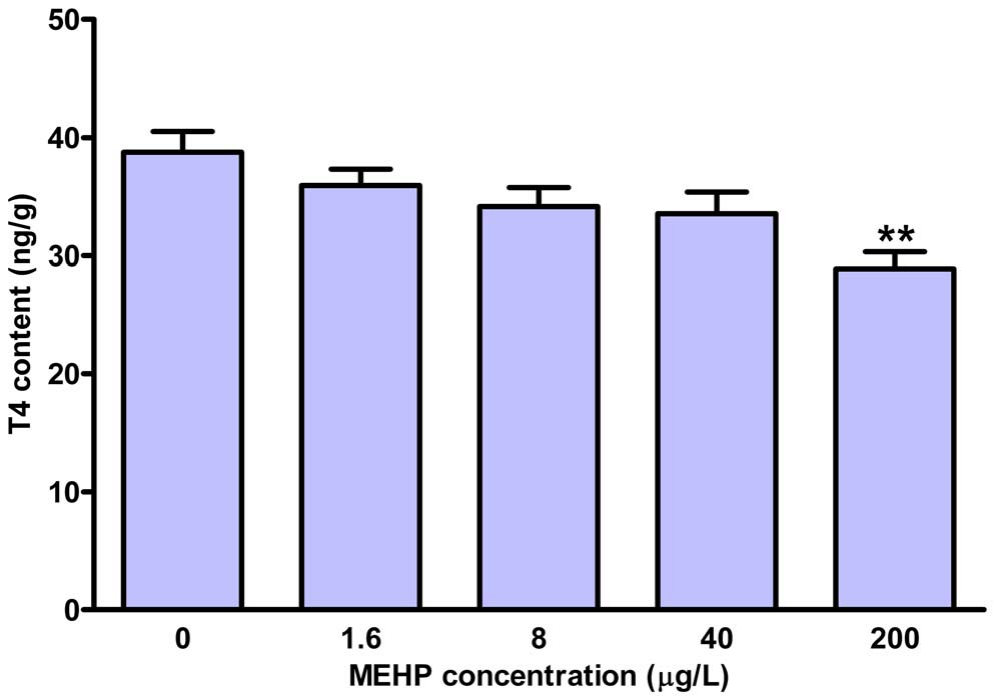
Whole-body thyroxine (T4) contents in zebrafish larvae exposed to different concentrations of MEHP until 168 hpf. Values represent mean±SEM (n = 6). Significant difference from the control group is indicated by ^**^ *P*<0.01.

**Figure 2:**
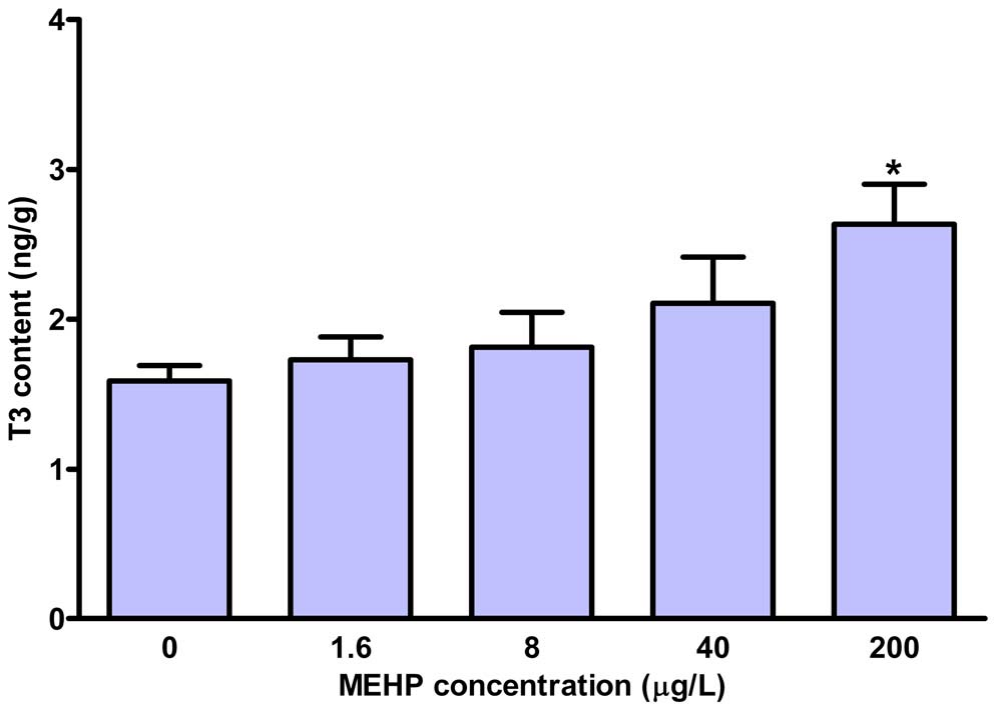
Whole-body triiodothyronine (T3) contents in zebrafish larvae exposed to different concentrations of MEHP until 168 hpf. Values represent mean±SEM (n = 6). Significant difference from the control group is indicated by ^*^ *P*<0.05.

The whole-body TH contents were measured in the larvae at 168 hpf. As shown in **Fig. 1**, MEHP exposure caused a concentration-dependent reduction in whole-body T4 contents. The total T4 contents were significantly decreased by 25.5% in 200 μg/L exposure group relative to the control. However, we observed an increase of total T3 contents in the exposure groups, which was significant in the 200 μg/L MEHP group (63.4%) **(** **Fig. 2** **)**.

### Gene transcription profile

**Figure 3:**
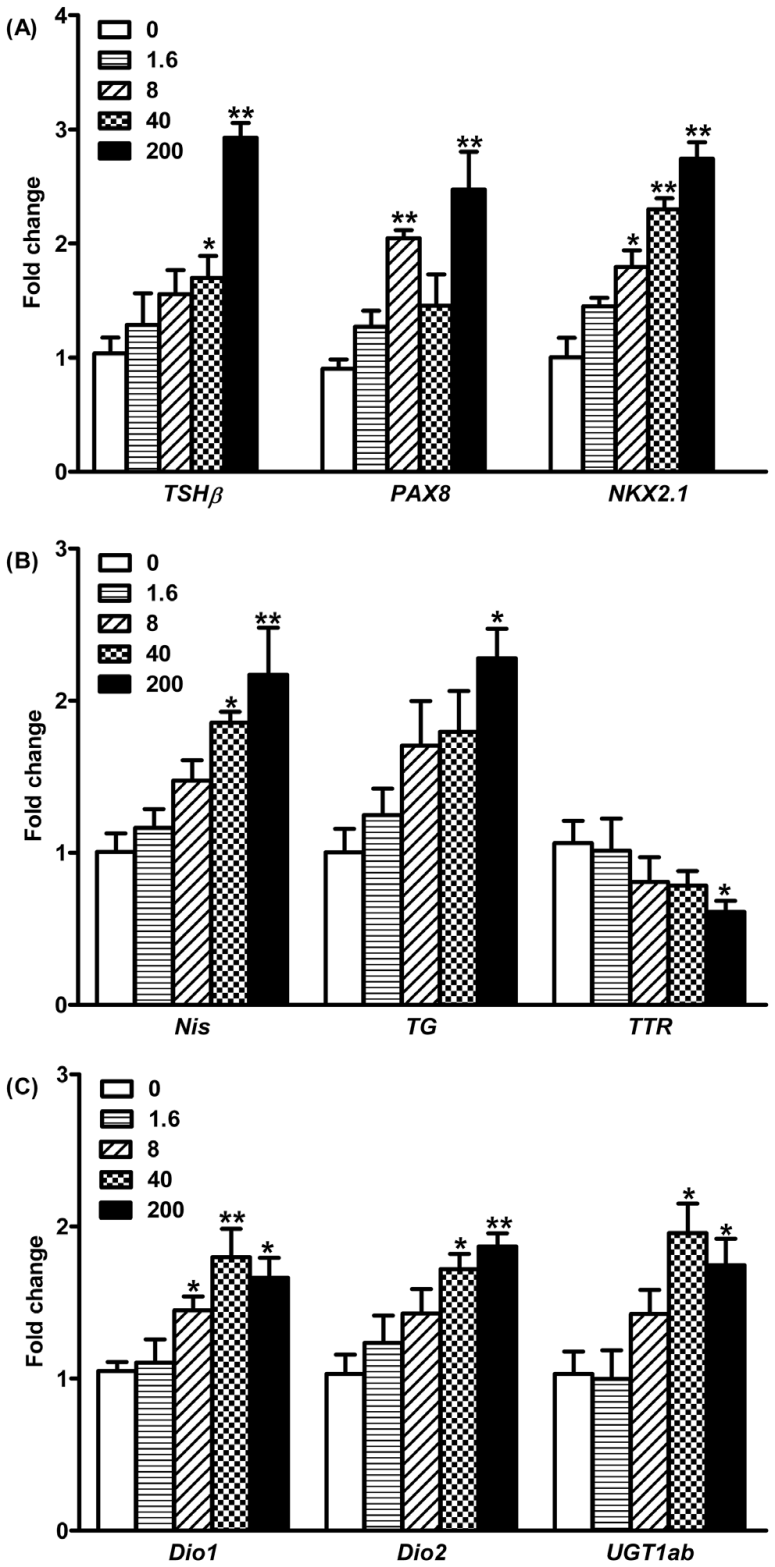
Gene transcription in hypothalamic-pituitary-thyroid (HPT) axis in zebrafish larvae after exposure to different concentrations of MEHP until 168 hpf. Data expressed as mean±SEM (n = 6). Significant difference from the control group is indicated by ^*^ *P*<0.05, and ^**^ *P*<0.01.

Several genes involved in the HPT axis of zebrafish were examined in this study. The transcription of the thyroid stimulating hormone (*TSHβ*) gene was significantly increased by 1.7- and 2.9-fold after exposure to 40 and 200 μg/L MEHP as compared to that in the control **(** **Fig. 3A** **)**. In identifying the marker genes involved in thyroid growth and development, *Pax8* was transcriptionally significantly induced by 2.0- and 2.5-fold in the 8 and 200 μg/L MEHP exposure groups, respectively **(** **Fig. 3A** **)**, while *Nkx2.1* was up-regulated by 1.8-, 2.3-, and 2.7-fold in a concentration-dependent manner after exposure to 8, 40, and 200 μg/L MEHP **(** **Fig. 3A** **)**. The mRNA expression of gene encoding sodium/iodide symporter (*NIS*) was significantly increased 1.9- and 2.2-fold after treatment with 40 and 200 μg/L MEHP, respectively **(** **Fig. 3B** **)**. Upon treatment with 200 μg/L MEHP, the thyroglobulin (*TG*) was significantly up-regulated transcriptionally by 2.3-fold **(** **Fig. 3B** **)**. However, the transthyretin (*TTR*) gene transcription was significantly down-regulated 1.7-fold after exposure to 200 μg/L MEHP **(** **Fig. 3B** **)**. In this study, two deiodinase isoforms (*Dio1* and *Dio2*) have been examined. The transcription of *Dio1* gene was significantly induced by 1.4-, 1.8-, and 1.7-fold in the 8, 40, and 200 μg/L MEHP groups, respectively **(** **Fig. 3C** **)**, while the mRNA expression of *Dio2* after treatment with 40 and 200 μg/L of MEHP was significantly increased 1.7- and 1.9-fold, respectively **(** **Fig. 3C** **)**. In addition, the gene involved in the metabolism of TH (uridinediphosphate glucoronosyltransferases, *UGT1ab*) was transcriptionally significantly up-regulated by 2.0- and 1.7-fold after exposure to 40 and 200 μg/L of MEHP, respectively **(** **Fig. 3C** **)**.

## Discussion

Studies exploring the association between MEHP exposure and thyroid endocrine disrupting effects are limited, however, growing human studies have indicated that MEHP has the potential to alter thyroid hormone levels [28]–[30]. In the present study, developing zebrafish embryos/larvae were employed to assess effects of MEHP on thyroid hormone contents (T4 and T3) and mRNA expression related to thyroid hormones synthesis, secretion, transport and metabolism. The results showed that MEHP changed whole-body thyroid hormone (T4 and T3) contents and expression of genes involved in the HPT axis, thus clearly demonstrating its thyroid endocrine disrupting activity.

It has been suggested that MEHP, the active metabolites of DEHP, is related to the toxic effects of its parent compound [2]. In the present study, a significant decrease in T4 contents and increase in T3 contents were observed with MEHP exposure. This result was, to a certain extent, in agreement with previous findings, in which oral exposure to DEHP was found to decrease the plasma T4 levels in rats [23], [41]. On the contrary, the serum T4 levels were increased in rats after intravenously receiving DEHP [27]. The inconsistent responses reported in these studies are likely due to different exposure scenarios and concentrations. A recent epidemiological study also demonstrated that increased urinary concentrations of MEHP were negatively correlated with free T4 concentrations in the serum of adult men [29], which is consistent with the results of our study.

The pituitary gland regulates thyroid activity through the secretion of TSH, and in turn TSH secretion is triggered by changes in the concentrations of circulating THs via feedback mechanisms [42]. TSH is a glycoprotein complex that composed of two subunits (α and β), and the β subunit (TSHβ) plays pivotal roles in the HPT axis. It has been proposed that assessment of *TSHβ* gene transcription can be used to determine whether environmental pollutants give rise to thyroid dysfunction. In the present study, transcription of *TSHβ* gene was significantly up-regulated after MEHP treatment. Reductions of T4 and accompanying up-regulation of *TSHβ* gene transcription have been previously observed in fathead minnows (*Pimephales promelas*) and zebrafish larvae after chemical exposure [40], [43], [44].

We also examined genes involved in thyroid growth and development (*Nkx2.1* and *Pax8*) and TH synthesis (*NIS* and *TG*) in the present study. Exposure to MEHP significantly increased transcription of *Nkx2.1* and *Pax* genes. It has been demonstrated that *Nkx2.1* (*TTF1*) and *Pax8* are the main transcription factors regulating the expression of *NIS* and *TG* in the thyroid system [45], [46]. Therefore, elevated gene transcription of *Nkx2.1* and *Pax8* may give rise to increased *NIS* and *TG*, which is in accordance with our findings. Similar results have also been observed for zebrafish embryos/larvae exposed to various pollutants [40], [44], [47].

TTR, an important transport protein for THs, plays an important role in the thyroid axis in fish by regulating the supply of the hormone to various target tissues [48]. In this study, MEHP exposure led to decrease of *TTR* gene transcription, which may reduce the amount of TTR binding to free THs. Consequently, the excess uncombined T4 would be more susceptible to hepatic catabolism, resulting in a greater elimination and a reduction in circulating TH concentrations [49].

Deiodinases play a key role in the regulation of circulating and peripheral TH levels in vertebrates. In teleosts, there are three types of deiodinases: type 1 (Dio1), type 2 (Dio2), and type 3 (Dio3), and each of them has different functions. Dio1 has a considerable influence on iodine recovery and thyroid hormone degradation [50]. Dio2 exclusively catalyzes outer-ring deiodination of T4 into active T3, consequently controls the intracellular concentration of T3, and Dio3 is a purely inactivating enzyme [51]. In this study, both *Dio1* and *Dio2* were significantly induced after MEHP exposure, consistent with previous study demonstrating that hypothyroidism increases Dio1 and Dio2 activities and mRNA expression [51]. Combining with previous studies, we suggest that induction in the transcription of *Dio2* may be, at least partly, responsible for the reduction of T4 contents, and increased transcription of *Dio1* may assist to degrade the elevated T3 contents as a compensatory mechanism. Besides deiodinases, uridinediphosphate glucuronosyltransferase (UGT) also plays an important role in TH metabolism, via the major pathway for T4 conjugation [52]. Previous studies conducted on rats and zebrafish have demonstrated that T4 concentrations were negatively associated with UGT activities or gene transcription after chemicals treatment [38], [40], [47], [53]–[55]. In agreement with these previous studies, a significant induction of *UGT1ab* mRNA expression was also observed in our study. This negative association between whole-body T4 contents (reduction) and *UGT1ab* expression (induction) suggests that UGT1ab might play a role in reducing circulating T4 concentrations.

In conclusion, our results demonstrated that exposure of MEHP to zebrafish larvae decreased the T4 contents but increased the T3 contents by changing a series of genes transcription involved in the HPT axis. These results clearly showed that MEHP has the potential to disrupt thyroid endocrine system in fish. Furthermore, the observed effective concentrations of MEHP in causing thyroid endocrine-disrupting activity in this study were several orders of magnitude greater than those reported in water [10]. Therefore, MEHP alone at concentrations commonly found in environment and during short-term exposures may not cause thyroid endocrine disrupting effects in fish species. To gain more complete toxicological profiles of MEHP, further research are require to examine the effects on adult fish and a long-term exposure. Taking into account the extensive use of DEHP and its biotransformation to MEHP, the thyroid endocrine toxicity of MHEP should be paid for more attention.
